# On optimal two‐stage testing of multiple mediators

**DOI:** 10.1002/bimj.202100190

**Published:** 2022-04-14

**Authors:** Vera Djordjilović, Jesse Hemerik, Magne Thoresen

**Affiliations:** ^1^ Department of Economics Ca' Foscari University of Venice Dorsoduro Venice Italy; ^2^ Biometris Wageningen University & Research Wageningen The Netherlands; ^3^ Oslo Centre for Biostatistics and Epidemiology Department of Biostatistics University of Oslo Blindern Oslo Norway

**Keywords:** familywise error rate, high‐dimensional mediation, multiple testing, partial conjunction hypothesis, screening

## Abstract

Mediation analysis in high‐dimensional settings often involves identifying potential mediators among a large number of measured variables. For this purpose, a two‐step familywise error rate procedure called ScreenMin has been recently proposed. In ScreenMin, variables are first screened and only those that pass the screening are tested. The proposed data‐independent threshold for selection has been shown to guarantee asymptotic familywise error rate. In this work, we investigate the impact of the threshold on the finite‐sample familywise error rate. We derive a power maximizing threshold and show that it is well approximated by an adaptive threshold of Wang et al. (2016, arXiv preprint arXiv:1610.03330). We illustrate the investigated procedures on a case‐control study examining the effect of fish intake on the risk of colorectal adenoma. We also apply our procedure in the context of replicability analysis to identify single nucleotide polymorphisms (SNP) associated with crop yield in two distinct environments.

## 1 INTRODUCTION

Mediation analysis is an important tool for investigating the role of intermediate variables lying on the path between an exposure or treatment (X) and an outcome variable (Y) (VanderWeele, 2015). Recently, mediation analysis has been of interest in emerging fields characterized by an abundance of experimental data. In genomics and epigenomics, researchers search for potential mediators of lifestyle and environmental exposures on disease susceptibility (Richardson et al., 2019); examples include mediation by DNA methylation of the effect of smoking on lung cancer risk (Fasanelli et al., 2015) and of the protective effect of breastfeeding against childhood obesity (Sherwood et al., 2019). In neuroscience, researchers search for the parts of the brain that mediate the effect of an external stimulus on the perceived sensation (Chén et al., 2017; Woo et al., 2015). In these and other problems of this kind, researchers wish to investigate a large number of putative mediators, with the aim of identifying a subset of relevant variables to be studied further. This problem has been recognized as transcending the traditional confirmatory causal mediation analysis and has been termed *exploratory mediation analysis* (Serang et al., 2017).

Within the hypothesis testing framework, the problem of identifying potential mediators among m variables $M_{i}$, i=1,…,m, can be formulated as the problem of testing a collection of m union hypotheses of the form

(1) $$H_{i}=H_{i1}\cup H_{i2},\;H_{i1}:M_{i}⊥\;\;\;\;⊥X,\;H_{i2}:M_{i}⊥\;\;\;\;⊥Y|{\left(X,M_{-i}\right)}^{⊤},$$

where $M_{-i}=\left(M_{1},\text{…},M_{i-1},M_{i+1},\text{…},M_{m}\right)$. Since m is typically large with respect to the study sample size, it might be challenging to make inference on the conditional independence of $M_{i}$ and Y given X and the entire (m−1)‐dimensional vector $M_{-i}$. To circumvent this issue, researchers often perform exploratory analysis in which each putative mediator is considered marginally (Sampson et al., 2018). In that case, $H_{i2}$ is formulated as $M_{i}⊥\;\;\;\;⊥Y|X$. The goal is to reject as many false union hypotheses $H_{i}$ as possible while keeping the familywise error rate below a prescribed level α∈(0,1), and this is the problem that we address in this article.

Assume we have valid p‐values, $p_{ij}$, for testing hypotheses $H_{ij}$. They would typically be obtained from two parametric models: a *mediator model* that models the relationship between X and M, and an *outcome model* that models the relationship between Y and X and M. Then, according to the intersection union principle, ${\bar{p}}_{i}=\max\left\{p_{i1},p_{i2}\right\}$ is a valid p‐value for $H_{i}$ (Gleser, 1973). A simple solution to the considered problem consists of applying a standard multiple testing procedure, such as Bonferroni or Holm (1979), to a collection of m maximum p‐values $\left\{{\bar{p}}_{i},\;i=1,\text{…},m\right\}$. Unfortunately, due to the composite nature of the considered null hypotheses, ${\bar{p}}_{i}$ will be a conservative p‐value for some points of the null hypothesis $H_{i}$. For instance, when both $H_{i1}$ and $H_{i2}$ are true, ${\bar{p}}_{i}$, will be distributed as the maximum of two independent standard uniform random variables, and thus stochastically larger than the standard uniform. As a consequence, the direct approach tends to be very conservative in most practical situations. Indeed, when only a small fraction of hypotheses $H_{ij}$ is false, which is a plausible assumption in most applications considered above, the actual familywise error rate can be shown to be well below α (Wang et al., 2016), resulting in a low‐powered procedure.

To attenuate this issue, we have recently proposed a two‐step procedure, ScreenMin, in which hypotheses are first screened on the basis of the minimum, ${\underline{p}}_{i}=\min\left\{p_{i1},p_{i2}\right\}$, and only hypotheses that pass the screening are tested:

Procedure 1 (ScreenMin (Djordjilović et al., 2019)) For a given c∈(0,1), select $H_{i}$ if ${\underline{p}}_{i}\leq c$, and let $S=\{i:{\underline{p}}_{i}\leq c\}$ denote the selected set. The ScreenMin adjusted p‐values are

(2) $$p_{i}^{*}=\left\{\begin{array}{c} \min\left\{\left|S\right|\;{\bar{p}}_{i},1\right\}\;\;\text{if}\;i\in S,\\ 1\;\text{otherwise,} \end{array}\right.$$

where |S| is the size of the selected set.

In other words, ScreenMin is a procedure with two thresholds, a screening threshold c, set by the user, and a testing threshold α/|S|, which is a function of the (random) number of hypotheses that pass the screening. It has been proved that, under the assumption of independence of all p‐values, the ScreenMin procedure provides asymptotic familywise error rate control, while significantly increasing the power to reject false union hypotheses. The recommended default threshold for screening is c=α/m (Djordjilović et al., 2019).

In this work, we investigate the crucial role of the threshold. Clearly, there is an inherent trade‐off associated to c: low values lead to fewer hypotheses passing the screening and a reduced multiplicity issue in the testing stage. On the other hand, since only hypotheses that pass the screening are tested, low values of c also reduce the number of hypotheses that can be rejected. Here, we try to answer a question of how should one choose c to balance out this trade‐off and maximize the power to reject false hypotheses. We show that the optimal value of c depends on the characteristics of the data distribution, that are often at least partially unknown. We thus introduce a data‐dependent threshold that in practice approximates the optimal threshold very well.

We start by showing that the ScreenMin procedure does not guarantee nonasymptotic familywise error rate control for all thresholds c∈(0,1). We derive the upper bound for the finite‐sample familywise error rate, and then investigate the optimal threshold, where optimality is defined in terms of maximizing the power while guaranteeing the finite‐sample familywise error rate control. We formulate this problem as a constrained optimization problem. The original problem requires optimizing the expected value of a nonlinear function of |S|, we thus resort to an approximation and solve it under the assumption that the proportion of false hypotheses and the distributions of the nonnull p‐values are known. We show that the solution is the smallest threshold that satisfies the familywise error rate constraint, and that the data‐dependent version of this oracle threshold leads to a special case of an adaptive threshold proposed recently in the context of testing general partial conjunction hypotheses by Wang et al. (2016). In their work, Wang et al. (2016) show that the proposed heuristic threshold guarantees familywise error rate control; our results provide further theoretical justification by showing that it is also (nearly) optimal in terms of power.

Recently, methodological issues pertaining to high‐dimensional mediation analysis have received increasing attention in the literature. Most proposed approaches focus on dimension reduction (Chén et al., 2017; Huang and Pan, 2016) or penalization techniques (Song et al., 2018; Zhang et al., 2016; Zhao and Luo, 2016), or a combination of the two (Zhao et al., 2020). The approach most similar to ours is a multiple testing procedure proposed by Sampson et al. (2018). The authors adapt to the mediation setting the procedures proposed by Bogomolov and Heller (2018) within the context of replicability analysis. Indeed, since the problem of identifying replicable findings across two independent studies can be formulated as a problem of testing multiple partial conjunction hypotheses (Benjamini & Heller, 2008), our procedure can be applied in this setting as well. As an illustration of a replicability analysis, we apply our method to crop trial data, to identify genetic loci in maize that are associated with yield in two distinct environments. We also apply our method in a classical mediation setting to identify metabolites acting as potential mediators of the protective effect of fish intake on the risk of colorectal adenoma. Data and code for reproducing all reported results are provided as Supplementary material available online.

## 2 NOTATION AND SETUP

As already stated, we consider a collection H of m null hypotheses of the form $H_{i}=H_{i1}\cup H_{i2}$. For each hypothesis pair $\left(H_{i1},H_{i2}\right)$, there are four possible states, {(0,0),(0,1),(1,0),(1,1)}, indicating whether respective hypotheses are true (0) or false (1). Let $\pi_{0}$ denote the proportion of (0,0) hypothesis pairs, that is, pairs in which both component hypotheses are true; $\pi_{1}$ the proportion of (0,1) and (1,0) pairs in which exactly one hypothesis is true, and $\pi_{2}$ the proportion of (1,1) pairs in which both hypotheses are false. In mediation, (1,1) hypotheses are of interest, and our goal is to reject as many such hypotheses as possible, while controlling familywise error rate for H.

We denote by $p_{ij}$ the p‐value for $H_{ij}$ and whether we refer to a random variable or its realization will be clear from the context. We assume that the $p_{ij}$ are continuous and independent random variables. We further assume that the distribution of the null p‐values is standard uniform, that the density of the nonnull p‐values is strictly decreasing, and that F denotes its cumulative distribution function. This will hold, for example, when the test statistics are normally distributed with a mean shift under the alternative; we will use this setting for illustration purposes throughout. We further let ${\bar{p}}_{i}$ (${\underline{p}}_{i}$) denote the maximum (the minimum) of $p_{i1}$ and $p_{i2}$.

For a given threshold c∈(0,1), let the selection event be represented by a vector $G=\left(G_{1},\text{…},G_{m}\right)\in {\left\{0,1\right\}}^{m}$, so that $G_{i}=1$ if ${\underline{p}}_{i}\leq c$ and $G_{i}=0$ otherwise. The size of the selected set is then $\left|S\right|=\sum_{j=1}^{m}G_{j}$.

## 3 FINITE‐SAMPLE FAMILYWISE ERROR RATE

Validity of the ScreenMin procedure relies on the maximum p‐value, ${\bar{p}}_{i}$, remaining an asymptotically valid p‐value after selection. We are thus interested in the distribution of ${\bar{p}}_{i}$ conditional on the selection G. We first look at the distribution of ${\bar{p}}_{i}$ conditional on the event that the ith hypothesis has been selected.

Lemma 1 If $\left(H_{i1},H_{i2}\right)$ is a (0,1) or a (1,0) pair, then the distribution of ${\bar{p}}_{i}$ conditional on hypothesis $H_{i}$ being selected is

(3) $$P\left({\bar{p}}_{i}\leq u|{\underline{p}}_{i}\leq c\right)=\left\{\begin{array}{c} \frac{uF(u)}{F(c)+c-cF(c)},\;\;\text{for}\;0<u\leq c\leq 1\\ \frac{cF(u)+uF(c)-cF(c)}{F(c)+c-cF(c)},\;\;\text{for}\;0<c\leq u\leq 1. \end{array}\right.$$

If $\left(H_{i1},H_{i2}\right)$ is a (0,0) pair, then

(4) $$P\left({\bar{p}}_{i}\leq u|{\underline{p}}_{i}\leq c\right)=\left\{\begin{array}{c} \frac{u^{2}}{c(2-c)},\;\;\text{for}\;0<u\leq c\leq 1\\ \frac{2u-c}{2-c},\;\;\text{for}\;0<c\leq u\leq 1. \end{array}\right.$$

The proof is in Section A.1. The p‐value in (3) will play an important role in the following considerations. Since it is a function of both the selection threshold c and the testing threshold u, we will denote it by $P_{0}\left(u,c\right)$.

Consider now the distribution of ${\bar{p}}_{i}$ conditional on the entire selection event G (where we are only interested in selections for which $G_{i}=1$). Given the independence of all p‐values,

(5) $$P\left({\bar{p}}_{i}\leq u|G\right)=P\left({\bar{p}}_{i}\leq u|G_{i}\right)=P_{0}\left(u,c\right)$$

for any fixed u∈(0,1). However, in the ScreenMin procedure we are not interested in all u; we are interested in a data‐dependent threshold α/|S|. Nevertheless, we can still use expression (3), since

(6) $$P\left({\bar{p}}_{i}\leq \frac{\alpha }{\left|S\right|}|G\right)=P\left({\bar{p}}_{i}\leq \frac{\alpha }{1+\sum_{j\neq i}G_{j}}|I\left[{\underline{p}}_{i}\leq c\right],\sum_{j\neq m}G_{j}\right)=P_{0}\left(\frac{\alpha }{\left|S\right|},\;c\right),$$

where the first equality follows from observing that when the ith hypothesis is selected we can write $\left|S\right|=1+\sum_{j\neq i}G_{j}$; and the second from the independence of ${\bar{p}}_{i}$ and $\sum_{j\neq i}G_{j}$.

Screening on the basis of the minimum ${\underline{p}}_{i}$, would ideally leave ${\bar{p}}_{i}$ a valid p‐value. Recall that a random variable is a valid p‐value if its distribution under the null hypothesis is either standard uniform or stochastically greater than the standard uniform. For a given c, for the p‐value in (3), we should thus have $P_{0}\left(u,c\right)\leq u$ for u∈(0,1). Although this has been shown to hold asymptotically (Djordjilović et al., 2019), the following analytical counterexample shows this might fail to hold in finite samples.

**FIGURE 1:**
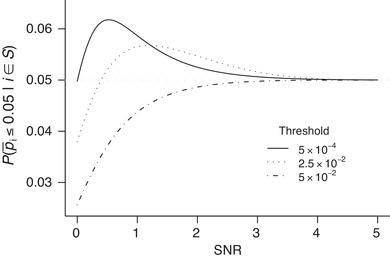
Conditional p‐value of the true union hypothesis: 5% quantile as a function of a signal‐to‐noise ratio of a possibly false component hypothesis. Solid, dotted, and dot‐dash curves correspond to the threshold $c=5\times 10^{-4},2.5\times 10^{-2}$, and $5\times 10^{-2}$, respectively. Dotted horizontal line y=0.05 is added for reference

Example 1 Let $H_{i}$ be true, and let the test statistics for testing $H_{i1}$ and $H_{i2}$ be normal with a zero mean and a mean in the interval [0,5], respectively, with unit variance. We refer to the mean shift associated to $H_{i2}$ as the signal‐to‐noise ratio (SNR). Figure 1 plots a 5% quantile of the conditional p‐value distribution, $P_{0}\left(0.05,c\right)$, as a function of the SNR associated to $H_{i2}$ for three different values of $c\in \{5\times 10^{-4},2.5\times 10^{-2},5\times 10^{-2}\}$. These values of c correspond to a default ScreenMin procedure with α=0.05 and m=100,2,1, respectively. Although with increasing SNR the quantile under consideration converges to 0.05 (in line with the asymptotic ScreenMin validity), for small values of SNR and low selection thresholds c, the conditional quantile surpasses 0.05.

According to Example 1 and expression (6), there are realizations of |S| so that $P_{0}\left(\alpha /|S|,c\right)$ is not bounded by α/|S|. This implies that the ScreenMin procedure will not always guarantee finite‐sample familywise error rate control *conditional* on |S|; however, it could still guarantee familywise error rate control *on average* across all |S|. To investigate this hypothesis, we first derive the upper bound for the unconditional familywise error rate for a given c. Proof is in Section A.2.

Proposition 1 Let V denote the number of true union hypotheses rejected by the ScreenMin procedure. For the familywise error rate, we then have

(7) $$P\left(V\geq 1\right)\leq E\left(\left[1-{\left\{1-P_{0}\left(\frac{\alpha }{\left|S\right|},c\right)\right\}}^{\left|S\right|}\right]I\left[\left|S\right|>0\right]\right),$$

with equality holding if and only if $\pi_{1}=1$.

We use this result to illustrate in the following analytical counterexample that ScreenMin does not guarantee unconditional finite‐sample familywise error rate control for arbitrary thresholds.

Example 2 Let m=10, and let all pairs $\left(H_{i1},H_{i2}\right)$ be (0,1) or (1,0) type, so that $\pi_{0}=\pi_{2}=0$ and $\pi_{1}=1$. Let the test statistics of all false $H_{ij}$ be normal with mean 2 and variance 1, and consider one‐sided p‐values. If the level at which familywise error rate is to be controlled is α=0.05, the default ScreenMin threshold for selection is $c=\alpha /m=5\times 10^{-3}$. The probability of selecting $H_{i}$ is then $P_{sel}=F\left(c\right)+c-cF\left(c\right)\approx 0.29$. In this case, the size of the selected set is a binomial random variable $\mathrm{Bi}(m,P_{sel})$. The conditional probability of rejecting a $H_{i}$ when |S|>0, that is, $P_{0}(\alpha /|S|,c)=P({\bar{p}}_{i}\leq \alpha /\left|S\right||I\left[{\underline{p}}_{i}\leq c\right],|S|)$, can be evaluated for each value of |S| according to (3). The conditional distribution of the number of false rejections V given |S| is also binomial with parameters |S| and $P_{0}\left(\alpha /|S|,c\right)$. In this case, the exact familywise error rate, obtained from (7), is Pr(V≥1)=0.055>α, so that the actual familywise error rate of the ScreenMin procedure exceeds the nominal level α.

## 4 ORACLE THRESHOLD FOR SELECTION

According to the previous section, not all thresholds for selection lead to finite‐sample familywise error rate control. In this section, we investigate the threshold that maximizes the power to reject false union hypotheses while ensuring finite‐sample familywise error rate control. The following proposition gives the power to reject a false union hypothesis conditional on the number of hypotheses that pass the screening.

Proposition 2 Let 1≤i≤m and suppose that $H_{i}$ is false. Then the probability of rejecting $H_{i}$ conditional on the size of the selected set |S| is

(8) $$P\left({\bar{p}}_{i}\leq \frac{\alpha }{\left|S\right|},{\underline{p}}_{i}\leq c\;|\left|S\right|\right)=\left\{\begin{array}{cc} 2F\left(c\right)F\left(\frac{\alpha }{\left|S\right|}\right)-F^{2}\left(c\right) & \text{for}\;c\;\left|S\right|\leq \alpha ;\\ F^{2}\left(\frac{\alpha }{\left|S\right|}\right) & \text{for}\;c\;\left|S\right|>\alpha \end{array}\right.$$

for |S|>0, and 0 otherwise. The unconditional probability of rejecting a false hypothesis is then obtained by taking the expectation over |S|.

See Section A.3 for the proof. Note that the distribution of S, as well as the distribution of V, depend on c, and in the following we emphasize this by writing S(c) and V(c). The threshold that maximizes the power while controlling familywise error rate at α can then be found through the following constrained optimization problem:

(9) $$\max_{0<c\leq \alpha }E\left[P\left({\bar{p}}_{i}\leq \frac{\alpha }{\left|S(c)\right|},\;{\underline{p}}_{i}\leq c\right)I\left[\left|S(c)\right|>0\right]\right]\;\text{subject}\;\text{to}\;P\left(V\left(c\right)\geq 1\right)\leq \alpha .$$

Both the objective function (the power) and the constraint (the familywise error rate) are expected values of nonlinear functions of the size of the selected set |S|, the distribution of which is itself nontrivial. To circumvent this issue, instead of (9), we consider its approximation based on the upper bound of Proposition 1 and exchanging the order of the function and the expected value:

(10) $$\max_{0<c\leq \alpha }P\left({\bar{p}}_{i}\leq \frac{\alpha }{E\left|S(c)\right|},\;{\underline{p}}_{i}\leq c\right)\;\text{subject}\;\text{to}\;\hat{P}\left(V\left(c\right)\geq 1\right)\leq \alpha ,$$

where

(11) $$\hat{P}\left(V\left(c\right)\geq 1\right)=1-{\left\{1-P_{0}\left(\frac{\alpha }{E\left|S(c)\right|},c\right)\right\}}^{E\left|S(c)\right|}.$$

When $\pi_{0},\pi_{1},\pi_{2}$, and F are known, (10) can be solved numerically. We denote its solution by $c^{*}$, and refer to it as the *oracle* threshold in what follows. We illustrate the constrained optimization problem of (10) in the following example.

**FIGURE 2:**
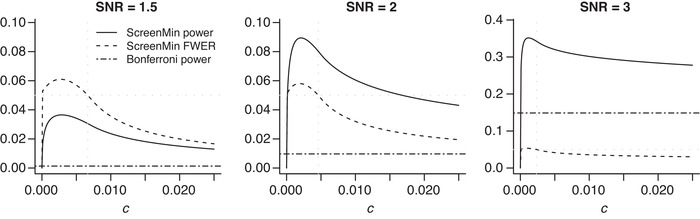
Approximated power and familywise error rate of the ScreenMin procedure as a function of c. Solid curve represents power; dashed curve represents familywise error rate. Dotted horizontal line y=0.05 represents the nominal familywise error rate. Dotted vertical line $x=c^{*}$ represents the oracle threshold, that is, the solution to the optimization problem (10). Dot‐dash line representing the power of the standard Bonferroni procedure is added for reference

Example 3 Consider an example featuring m=100 union hypotheses with proportions of different hypotheses being $\pi_{0}=0.7$, $\pi_{1}=0.25$, and $\pi_{2}=0.05$. Let the test statistics be normal with a zero mean for true null hypotheses and a mean shift (SNR) of 1.5,2, or 3 for false null hypotheses with variance equal to 1 in both cases. As before we consider one‐sided p‐values. Plots in Figure 2 show the approximated power and the constraint from (10) as functions of the selection threshold for three different values of the signal strength.

We first note that for very small values of c, the familywise error rate constraint is not satisfied. In all three cases, the value of the threshold that maximizes the unconstrained objective function is low and does not satisfy the constraint (dashed line is above the nominal familywise error rate level set to 0.05).

In the above example, the power maximizing selection threshold is the smallest threshold that satisfies the familywise error rate constraint. This can be shown to hold in general under mild conditions (see Section A.4 for details).

For a threshold to satisfy the familywise error rate constraint in (10), it needs to be at least as large as the solution to

(12) $$1-{\left\{1-P_{0}\left(\frac{\alpha }{E\left|S(c)\right|},c\right)\right\}}^{E\left|S(c)\right|}=\alpha .$$

If m is large, we can consider a first‐order approximation of the left‐hand side leading to

(13) $$P_{0}\left(\frac{\alpha }{E\left|S(c)\right|},c\right)\approx \frac{\alpha }{E\left|S(c)\right|}.$$

The intuition corresponding to (13) is straightforward: for a given c, the probability that a conditional null p‐value is less or equal to the “average” testing threshold, that is, α/E|S(c)|, should be exactly α/E|S(c)|. Finally, when m is large, the solution to (13) can be closely approximated by the solution to

(14) $$c\;E\left|S(c)\right|=\alpha$$

(see Section A.4) so that the constrained optimization problem in (10) can be replaced with a simpler problem of finding a solution to Equation (14).

## 5 ADAPTIVE THRESHOLD FOR SELECTION

Solving Equation (14) is easier than solving the constrained optimization problem of (10); however, it still requires knowing $F,\pi_{0}$, and $\pi_{1}$. To overcome this issue one can try to estimate these quantities from data in an approach similar to the one of Lei and Fithian (2018) who employ an expectation‐maximization algorithm.

Another possibility is to consider the following strategy. Instead of searching for a threshold optimal *on average*, we can adopt a *conditional* approach and replace E|S(c)| in (14) with its observed value S(c). Since S(c) takes on integer values, c|S(c)| has jumps at ${\underline{p}}_{1},\text{…},{\underline{p}}_{m}$ and might be different from α for all c. We therefore search for the largest c∈(0,1) such that

(15) $$c\;\left|S(c)\right|\leq \alpha .$$

Let $c_{a}$ be the solution to (15). This solution has been proposed in Wang et al. (2016) in the following form:

(16) $$\gamma =\max\left\{c\in \left\{\frac{\alpha }{m},\text{…},\frac{\alpha }{2},\alpha \right\}:c\;\left|S(c)\right|\leq \alpha \right\}.$$

Obviously, due to a finite grid, γ need not necessarily coincide with $c_{a}$; however, they lead to the same selected set S and thus to equivalent procedures. Interestingly, in their work, Wang et al. (2016) search for a single threshold that is used for both selection and testing, and define it heuristically as a solution to the above maximization problem. Their proposal is motivated by the observation that when the two thresholds coincide, $P_{0}\left(c,c\right)$ is bounded by c for all c∈(0,1) (from Equation 3), and it is straightforward to show that the familywise error rate control is maintained for the data‐dependent threshold c=γ. Our results show, that in addition to providing nonasymptotic familywise error rate control, this threshold is also nearly optimal in terms of power.

## 6 FINITE‐SAMPLE PER‐FAMILY ERROR RATE (PEFR)

So far we have focused on familywise error rate control. Other types of error quantification can also be of interest. For example, it is common to estimate the *false discovery rate*, which is the expected fraction of false positives among all findings (Storey, 2002). Similarly, one may want to simply estimate the expected *number* of false positive findings. We now show that this is possible in our setting.

Consider a data‐independent thresholds c∈(0,1) and suppose c is used for the selection in the first stage and as the threshold in the second stage. The expected number of false positive findings, E(V) is called the *per‐family error rate*. Considering the PFER can have certain advantages over only considering the familywise error rate (FWER), as discussed in, for example, Lawrence (2019). We have the following result.

Theorem 1 Define $\hat{PFER}=\left|S\left(c\right)\right|c$. Then $\hat{PFER}$ is an unbiased (or upward biased) estimate of E(V), that is,

(17) $$E\left(V\right)\leq E\left(\hat{PFER}\right).$$

The proof is provided in A.5.

To control (rather than only estimate) the PFER, we might choose c data‐dependently in such a way that $\hat{PFER}$ is low. In that case, the unique threshold for screening and testing

(18) $$c_{k}=\max\left\{c\in \left\{\frac{k}{m},\text{…},\frac{k}{2},k\right\}:c\;\left|S(c)\right|\leq k\right\}$$

ensures that PFER is bounded by k.

Theorem 2 Let $c_{k}$ in (18) be a data‐dependent threshold used for selection in the first stage and testing in the second stage. Then E(V)≤k.

The proof is provided in A.6.

## 7 SIMULATIONS

We used simulations to assess the performance of different selection thresholds. Our data‐generating mechanism is as follows. We considered a small, m=200, and a large, m=10,000, study. The proportion of false union hypotheses, $\pi_{2}$, was set to 0.05 throughout. The proportion of (1,0) hypothesis pairs with exactly one true hypothesis, $\pi_{1}$, was varying in {0,0.1,0.2,0.3,0.4}. Independent test statistics for false $H_{ij}$ were generated from $N(\sqrt{n}\mu_{j},1)$, where n is the sample size of the study, and $\mu_{j}>0$, j=1,2, is the effect size associated with false component hypotheses. Test statistics for true component hypotheses were standard normal. For m=200, the SNR, $\sqrt{n}\mu_{j}$, was either the same for j=1,2 and equal to 3, or different and equal to 3 and 6, respectively. For m=10,000, the SNR was set to 4, and in case of unequal SNR it was set to 4 and 8. p‐Values were one‐sided. Familywise error rate was controlled at α=0.05. We also considered settings under positive dependence: in that case the test statistics were generated from a multivariate normal distribution with a compound symmetry variance matrix with the correlation coefficient ρ∈{0.3,0.8} (results not shown).

The familywise error rate procedures considered were (1) ScreenMin procedure with the oracle threshold $c^{*}$ found as the solution to (10) assuming $F,\pi_{1},\pi_{2}$ to be known; (2) ScreenMin procedure with the adaptive threshold γ; (3) ScreenMin procedure with a default threshold c=α/m; 4) the familywise error rate procedure proposed in Sampson et al. (2018); and (5) the classical one stage Bonferroni procedure.

When applying the procedure of Sampson et al. (2018), we used the implementation in the MultiMed R package (Boca et al., 2018) with the default threshold $\alpha_{1}=\alpha_{2}=\alpha /2$. We note that the threshold for this procedure can also be improved in an adaptive fashion by incorporating plug‐in estimates of proportions of true hypotheses among $H_{i1}$, and $H_{i2}$, i=1,…,m, as presented in Bogomolov and Heller (2018). Implementation of the remaining procedures, along with the reproducible simulation setup, is available at http://github.com/veradjordjilovic/screenMin.

For each setting, we estimated familywise error rate as the proportion of generated data sets in which at least one true union hypothesis was rejected. We estimated power as the proportion of rejected false union hypotheses among all false union hypotheses, averaged across 1000 generated data sets.

**FIGURE 3:**
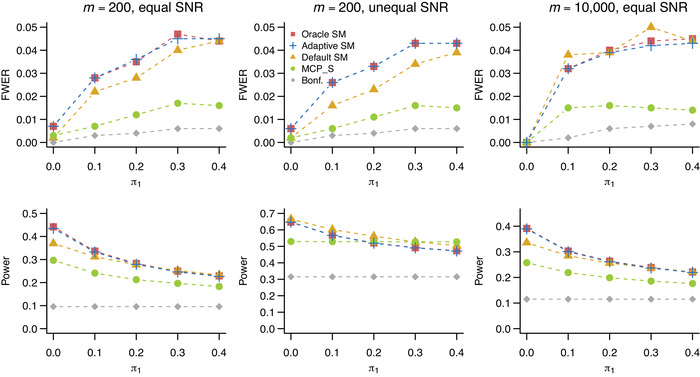
Estimated familywise error rate (first row) and power (second row) as a function of $\pi_{1}$ based on 1000 simulated data sets. The proportion of false union hypotheses is $\pi_{2}=0.05$. In columns 1 and 2: m=200, in column 3 m=10,000. Signal‐to‐noise ratio (SNR) is 3 for all false component hypotheses in column 1; 3 for $H_{i1}$ and 6 for $H_{i2}$ in column 2, 4 in column 3. Methods are ScreenMin with the oracle threshold (square), the adaptive threshold (cross), and the default threshold (triangle); the method of Sampson et al. (2018) (circle) and the classical Bonferroni (diamond). Monte Carlo standard errors of the estimates of power and familywise error rate are $1.6\times 10^{-2}$ and $7\times 10^{-3}$, respectively

Results under independence are shown in Figure 3. All considered procedures successfully control familywise error rate. When most hypothesis pairs are (0,0) pairs and $\pi_{1}$ is low, all procedures are conservative, but with increasing $\pi_{1}$ their actual familywise error rate approaches α. The opposite trend is seen with the power: it reaches its maximum for $\pi_{1}=0$ and decreases with increasing $\pi_{1}$. When the SNR is equal (columns 1 and 3), both ScreenMin with the oracle and adaptive threshold outperform the rest in terms of power. Interestingly, the adaptive threshold is performing as well as the oracle threshold which uses the knowledge of $F,\pi_{0}$, and $\pi_{1}$. Under unequal SNR, the oracle threshold is computed under a misspecified model (assuming the SNR is equal for all false hypotheses) and in this case the default threshold ScreenMin outperforms the other approaches. The procedure of Sampson et al. (2018) performs well in this setting and its power remains constant with increasing $\pi_{1}$.

**FIGURE 4:**
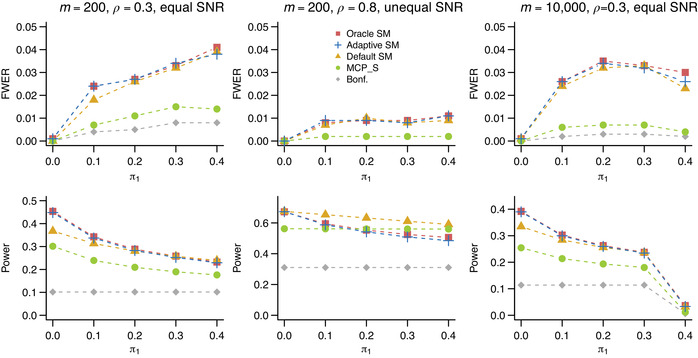
Estimated familywise error rate (first row) and power (second row) under dependence based on 1000 simulated data sets. Methods and signal to noise ratio are as in Figure 3

Results under positive dependence are shown in Figure 4. Familywise error rate control is maintained for all procedures. All procedures are more conservative in this setting than under independence, especially when the correlation is high, that is, when ρ=0.8. With regards to power, most conclusions from the independence setting apply here as well. When the SNR is equal, ScreenMin oracle and adaptive thresholds outperform competing procedures. Under unequal SNR, the default threshold performs best, and the procedure of Sampson et al. (2018) performs well with power constant with increasing $\pi_{1}$. In the high‐dimensional setting (m=10,000), the power is higher than under independence for $\pi_{1}=0$, but it is rapidly decreasing with increasing $\pi_{1}$ and drops to zero when $\pi_{1}=0.4$.

We further considered the following simulation setting. As before we set m=200, but now $\pi_{0}=0,\pi_{2}=0$, and $\pi_{1}=1$, so that all union hypotheses have exactly one false component hypothesis, and thus no union hypothesis is false. We varied the SNR in the range 3.1 and 3.9 and simulated 20,000 data sets. For each considered method, we estimated FWER and compared it with the target nominal rate of 5%. Table 1 displays the results.

**TABLE 1:** Estimated familywise error rate in percentages for the five methods: ScreenMin with the oracle threshold (Oracle SM), the adaptive threshold (Adaptive SM), the default threshold (Default SM), the method of Sampson et al. (2018) (MCP_S), and classical Bonferroni (Bonf)

SNR	Oracle SM	Adaptive SM	Default SM	MCP_S	Bonf
3.1	4.98	4.86	5.62	2.04	1.8
3.3	5	4.86	5.4	2.22	2.16
3.5	5	4.93	5.24	2.32	2.6
3.7	5.15	4.96	5.26	2.42	2.97
3.9	5.07	4.94	5.09	2.48	3.43

It is evident that in this setting ScreenMin with the default threshold exceeds the target error rate (the range of the estimated error rates is 5.09–5.62). This empirical result is in line with the theoretical result presented in Example 3.4 (with m=200). Note that the difference with respect to the previously considered settings is in the proportions $\pi_{0},\pi_{1}$, and $\pi_{2}$. The situation with $\pi_{1}=1$ is the worst‐case scenario for the default method.

The remaining methods maintain error control as expected. Interestingly, when the SNR is equal to 3.7 or 3.9, the Oracle ScreenMin method slightly exceeds the target error rate. This is likely due to an error of approximation employed when deriving the value of the optimal threshold (see Section 4).

## 8 APPLICATIONS

### 8.1 Navy Colorectal Adenoma study

The Navy Colorectal Adenoma case‐control study (Sinha et al., 1999) studied dietary risk factors of colorectal adenoma, a known precursor of colon cancer. A follow‐up study investigated the role of metabolites as potential mediators of an established association between red meat consumption and colorectal adenoma. While red meat consumption is shown to increase the risk of adenoma, it has been suggested that fish consumption might have a protective effect. In this case, the exposure of interest is daily fish intake estimated from dietary questionnaires; potential mediators are 149 circulating metabolites; and the outcome is a case‐control status. Data for 129 cases and 129 controls, including information on age, gender, smoking status, and body mass index, are available in the MultiMed R package (Boca et al., 2018).

For each metabolite, we estimated a mediator and an outcome model. The mediator model is a normal linear model with the metabolite level as outcome and daily fish intake as predictor. The outcome model is logistic with case‐control status outcome and fish intake and metabolite level as predictors. Age, gender, smoking status, and body mass index were included as predictors in both models. To adjust for the case‐control design, the mediator model was weighted on the basis of the prevalence of colorectal adenoma in the considered age group (0.228) reported in Boca et al. (2014).

Screening with a default ScreenMin threshold $0.05/149=3.3\times 10^{-4}$ leads to 13 hypotheses passing the selection. The adaptive threshold γ is higher ($2.2\times 10^{-3}$) and results in 22 selected hypotheses. The testing threshold for the default ScreenMin is then $0.05/13=3.8\times 10^{-3}$. With the adaptive procedure, the testing threshold coincides with the screening threshold and is slightly lower ($2.2\times 10^{-3}$). Unadjusted p‐values for the selected metabolites are shown in Table 2. The lowest maximum p‐value among the selected hypotheses is $8.3\times 10^{-3}$ (for DHA and 2‐aminobutyrate) which is higher than both considered thresholds, meaning that we are unable to reject any hypothesis at the α=0.05 level. Although we are unable to identify any potential mediators while controlling familywise error rate at 5%, if we instead consider a more lenient criterion of PFER and set k=1 (see Section 6), the obtained threshold $\gamma_{1}=2.2\times 10^{-2}$ results in rejecting four null hypotheses. In addition to three metabolites highlighted in Table 2, the null hypothesis of no mediation is rejected for 3‐hydroxyisobutyrate. Our results are in line with those reported in Boca et al. (2014), where the DHA was found to be the most likely mediator although not statistically significant (familywise error rate adjusted p‐value 0.06).

**TABLE 2:** p‐Values of the 22 metabolites that passed the screening with the adaptive threshold

	Name	$\underline{p}$	$\bar{p}$	Min.Ind
**1**	**2‐hydroxybutyrate (AHB)**	$1.2\times 10^{-6}$	$1.5\times 10^{-2}$	**2**
**2**	**docosahexaenoate (DHA; 22:6n3)**	$1.9\times 10^{-6}$	$8.3\times 10^{-3}$	**1**
3	3‐hydroxybutyrate (BHBA)	$7.8\times 10^{-6}$	$2.2\times 10^{-1}$	2
4	oleate (18:1n9)	$2.5\times 10^{-5}$	$7.3\times 10^{-1}$	2
5	glycerol	$3.9\times 10^{-5}$	$8.4\times 10^{-1}$	2
6	eicosenoate (20:1n9 or 11)	$5.9\times 10^{-5}$	$4.1\times 10^{-1}$	2
7	dihomo‐linoleate (20:2n6)	$9.0\times 10^{-5}$	$2.6\times 10^{-1}$	2
8	10‐nonadecenoate (19:1n9)	$9.4\times 10^{-5}$	$5.4\times 10^{-1}$	2
9	creatine	$1.7\times 10^{-4}$	$9.2\times 10^{-1}$	1
10	palmitoleate (16:1n7)	$1.7\times 10^{-4}$	$6.3\times 10^{-1}$	2
11	10‐heptadecenoate (17:1n7)	$2.8\times 10^{-4}$	$7.1\times 10^{-1}$	2
12	myristoleate (14:1n5)	$2.9\times 10^{-4}$	$8.2\times 10^{-1}$	2
13	docosapentaenoate (n3 DPA; 22:5n3)	$3.0\times 10^{-4}$	$2.9\times 10^{-1}$	2
14	methyl palmitate (15 or 2)	$5.4\times 10^{-4}$	$1.8\times 10^{-1}$	2
15	N‐acetyl‐beta‐alanine	$5.9\times 10^{-4}$	$1.3\times 10^{-1}$	1
16	linoleate (18:2n6)	$8.8\times 10^{-4}$	$6.7\times 10^{-1}$	2
17	3‐methyl‐2‐oxobutyrate	$8.9\times 10^{-4}$	$2.0\times 10^{-1}$	2
18	palmitate (16:0)	$9.9\times 10^{-4}$	$5.6\times 10^{-1}$	2
19	fumarate	$1.4\times 10^{-3}$	$5.0\times 10^{-1}$	2
**20**	**2‐aminobutyrate**	$1.4\times 10^{-3}$	$8.3\times 10^{-3}$	**2**
21	linolenate [alpha or gamma; (18:3n3 or 6)]	$1.6\times 10^{-3}$	$5.4\times 10^{-1}$	2
22	10‐undecenoate (11:1n1)	$1.8\times 10^{-3}$	$3.2\times 10^{-1}$	2

*Note*: Metabolites are sorted in an increasing order with respect to $\underline{p}$. The top 13 metabolites passed the screening with the default ScreenMin threshold. The last column (Min.Ind) indicates whether the minimum, $\underline{p}$, is the p‐value for the association of a metabolite with the fish intake (1) or with the colorectal adenoma (2). Metabolites for which the null hypothesis was rejected when target PFER was set to 1 are highlighted.

**FIGURE 5:**
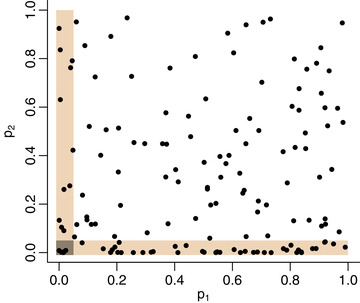
p‐Values for the association of 149 metabolites with the fish intake ($p_{1}$) and the risk colorectal adenoma ($p_{2}$). Each dot represents a single metabolite. Shaded area highlights p‐value pairs in which the minimum is below α=0.05

One potential explanation for the absence of significant findings at the level of 5% is illustrated in Figure 5. Figure 5 shows a scatterplot of the p‐values for the association of metabolites with the fish intake ($p_{1}$) against the p‐values for the association of metabolites with the colorectal adenoma ($p_{2}$). While a significant number of metabolites shows evidence of association with adenoma (cloud of points along the y=0 line), there seems to be little evidence for any association with fish intake. In addition, data provide limited evidence of the presence of any total effect of fish intake on the risk of adenoma (p‐value in the logistic regression model adjusted for age, gender, smoking status, and body mass index is 0.07). Findings reported in the literature regarding the effect of omega‐3 fatty acids, such as DHA, on adenoma risk, remain inconclusive. A protective effect was identified in a number of observational studies (Butler et al., 2009; Ghadimi et al., 2008; Song et al., 2014), the potential mechanism of action was investigated in Cockbain et al. (2012), but a recent intervention study (Song et al., 2020) found no effect of omega‐3 supplementation on reducing the risk of adenoma in the general population.

In this example, metabolites were considered one by one in the mediator and in the outcome model. Since metabolites are almost surely dependent even after adjusting for available potential confounders, these marginal models are likely misspecified. Nevertheless, they still prove useful in a preliminary exploratory analysis, such as the one reported here, since they allow us to identify potential mediators and greatly reduce the number of metabolites to be studied further in a joint model or by means of experimental methods.

### 8.2 Replicability of genome‐wide association study (GWAS) findings across two crop trials

In this section, we apply our method within the framework of replicability analysis to identify significant SNPs in two genomewide studies. Data that we consider are from a large multiyear, multilocation study of 256 maize hybrids (Millet et al., 2019) and are available in statgenGWAS R package (van Rossum and Kruijer, 2020).

**FIGURE 6:**
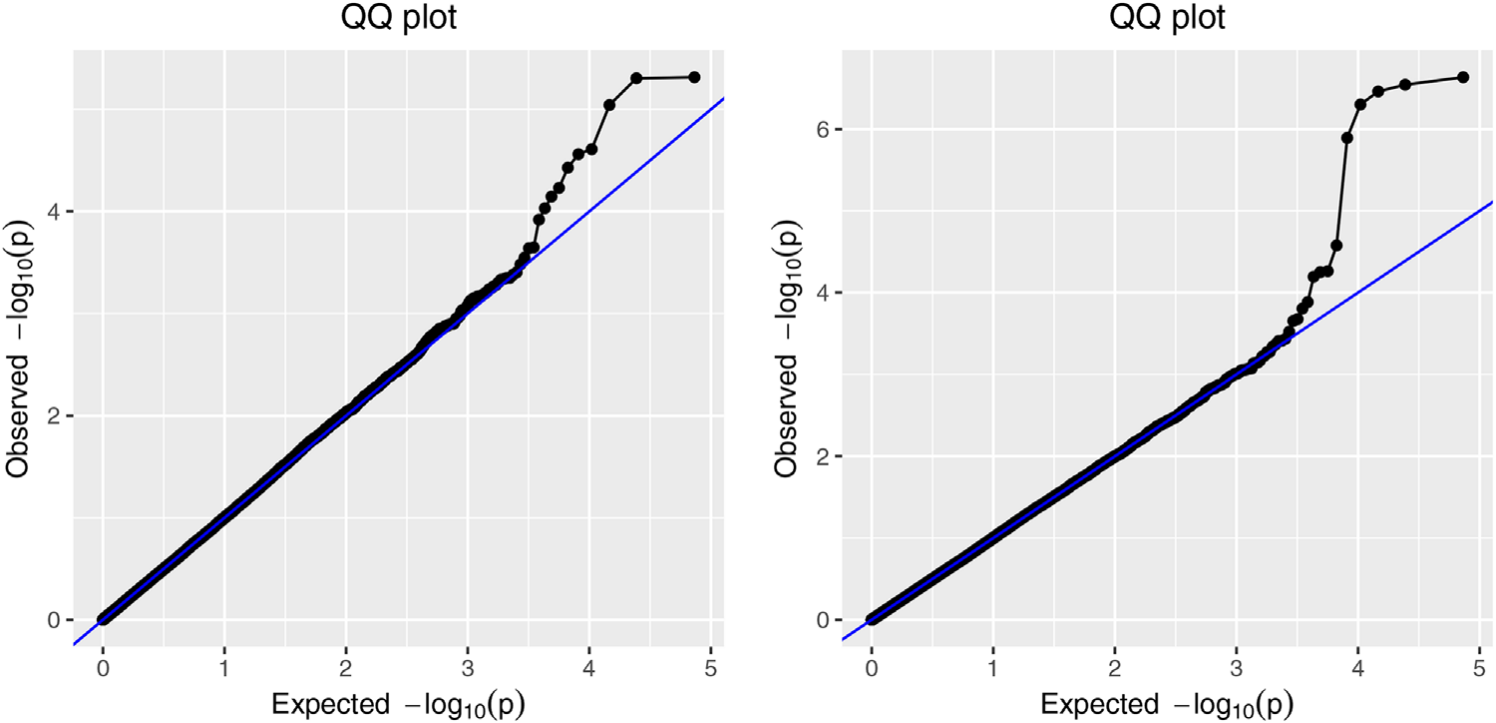
QQ‐plots of GWAS p‐values for Karlsruhe (left) and Murony (right)

We aimed to identify SNPs significantly associated to yield at two distinct environments considered in the study: in Karlsruhe in Germany and Murony in Hungary. Both fields were treated with the same treatment (“Watered”) and data are based on harvests from 2013. The analysis here is purely meant as an illustration, since we only use data from two trials from this multiyear, multilocation study (Figure 6).

After removing duplicates we were left with 36,624 SNPs. We performed two separate GWAS analyses (with the runSingleTraitGwas function of the statgenGWAS package) to compute p‐values for each SNP. All linear models include a random effect for genotype to account for population structure. For further details on the fitted linear models, we refer the interested reader to the statgenGWAS vignette.

With α=0.05, the ScreenMin default threshold is $1.36\times 10^{-6}$ and the adaptive threshold is $8.2\times 10^{-4}$. The default threshold results in five SNPs passing the screening, but none of the filtered SNPs passes the testing threshold, that is, all five adjusted p‐values are above 0.05. With the adaptive threshold, one SNP on chromosome 3 (id: PUT‐163a‐148986271‐678) and one on chromosome 4 (id: PZE‐104137686) have adjusted p‐values of $2.2\times 10^{-2}$ and $3.5\times 10^{-2}$, respectively. They are thus significant at the 5% level. On closer inspection, they are both strongly correlated with yield (Pearson correlation coefficient with yield in Karlsruhe and Murony of 0.33 and 0.31 for PZE‐104137686 and −0.31 and −0.36 for PZE‐104137686).

We further considered the adaptive threshold obtained when the target PFER is set to k=1. In this case, the threshold equals $3.7\times 10^{-3}$ and results in six additional significant SNPs. The results are reported in Table 3.

**TABLE 3:** Replicated SNPs when target PFER is set to 1

			Coefficient estimate		
	Name	Chromosome	Murony	Karlsruhe	$\bar{p}$
1	PZE‐101117779	1	0.54 (0.17)	0.77 (0.17)	$1.9\times 10^{-3}$
2	PZE‐101117823	1	0.52 (0.18)	0.76 (0.17)	$3.1\times 10^{-3}$
3	SYN2051	1	0.31 (0.10)	0.33 (0.10)	$2.4\times 10^{-3}$
4	PUT‐163a‐148986271‐678	3	0.47 (0.13)	0.59 (0.13)	$3.7\times 10^{-4}$
5	PZE‐104137686	4	−0.43 (0.12)	−0.39 (0.11)	$5.7\times 10^{-4}$
6	ZM013389‐0408	5	−0.36 (0.11)	−0.38 (0.11)	$9.8\times 10^{-4}$
7	SYN12761	8	−0.34 (0.11)	−0.37 (0.11)	$1.6\times 10^{-3}$
8	PZE‐108011901	8	0.39 (0.13)	0.37 (0.12)	$3.1\times 10^{-3}$

*Note*: Standard errors are reported in brackets; p‐values are unadjusted.

## 9 DISCUSSION

In this article, we have investigated power and nonasymptotic familywise error rate of the ScreenMin procedure as a function of the selection threshold. We have found an upper bound for the finite‐sample familywise error rate that is tight when $\pi_{1}=1$. We have posed the problem of finding an optimal selection threshold as a constrained optimization problem in which the approximated power to reject a false union hypothesis is maximized under the condition guaranteeing familywise error rate control. We have called this threshold the oracle threshold since it is derived under the assumption that the mechanism generating p‐values is fully known. We have shown that the solution to this optimization problem is the smallest threshold that satisfies the familywise error rate condition, and that it is well approximated by the solution to the equation cE|S(c)|=α. A data‐dependent version of the oracle threshold is a special case of the AdaFilter threshold proposed by Wang et al. (2016), for n=r=2 in their notation. Our simulation results suggest that the performance of this adaptive threshold is almost indistinguishable from the oracle threshold, and we suggest its use in practice.

The ScreenMin procedure relies on the independence of p‐values. While independence between columns in the p‐value matrix is satisfied in the context of mediation analysis (under correct specification of the mediator and the outcome model), independence within columns of the p‐value matrix is likely to be unrealistic in a number of practical contexts. A possible strategy to alleviate this issue is to adjust, when possible, mediator and outcome models for factors that are likely, at least partially, responsible for dependence among potential mediators. An example is given by the adjustment for population structure in GWAS models, as we consider in our application in Section 8.2. In addition, our simulation results show that familywise error rate control is maintained under mild and strong positive dependence within columns. The challenge with relaxing the independence assumption lies in the fact that when ${\bar{p}}_{i}$ is not independent of $\sum_{j\neq i}G_{j}$, the equality regarding conditional p‐values (6) no longer necessarily holds. Finding sufficient conditions that relax the assumption of independence while keeping the conditional distribution of p‐values tractable is an open question.

When screening a large number of potential mediators, researchers often consider them marginally. This choice is typically driven by the difficulty of the problem of high‐dimensional statistical inference (Goeman & Böhringer, 2020), in particular that of testing conditional independence of $M_{j}$ and Y given X and remaining m−1 potential mediators when m is large. Recently, two approaches that tackle this issue have been proposed. Chakrabortty et al. (2018) assumes that an unknown directed acyclic graph describes the relationship between the exposure, the mediators and the outcome and then extends the method IDA, previously proposed for identifying causal effects from observational data to identify newly defined individual mediation effects. In addition, the authors provide high‐dimensional consistency and distributional results for the proposed method, which can be employed to obtain asymptotic confidence intervals for the individual mediation effects. Shi and Li (2021) also assume a directed acyclic graphical structure, but introduce a slightly different definition of the individual mediation effect which circumvents the problem of disjunctive effects cancelling each other out and resulting in a zero mediation effect. The authors propose a novel method for testing mediation effects based on the logic of Boolean matrices, which allows taking into account directed paths among mediators, and still obtaining a tractable, limiting distribution of the test statistic under the null hypothesis. In addition, the authors combine the test statistic with the ScreenMin‐type screening to significantly improve power, while providing asymptotic type I error control.

Theoretical considerations leading to the optimal screening threshold are based on the assumption that the null p‐values are standard uniform. In practice, conservative tests might result in p‐values that are stochastically greater than the uniform distribution. In that case, the threshold derived will still guarantee finite‐sample error control, but might not be the threshold that maximizes the power. In other words, the conservativeness of p‐values will translate to conservativeness of the ScreenMin procedure.

Further important assumption underlying the optimality results presented in this work is that all nonnull p‐values have the same distribution F. In practice, associations between the exposure and mediators can be generally stronger (or weaker) than those between mediators and the outcome. Results presented here can be extended to this setting by introducing two distinct distributions $F_{1}$ and $F_{2}$ pertaining to the false hypotheses among $H_{i1}$ and $H_{i2}$, i=1,…,m, respectively. However, more importantly, the proposed adaptive threshold does not rely on any assumption regarding the distribution of the nonnull p‐values.

In this work, we have focused on familywise error rate, but it is tempting to consider combining screening based on ${\underline{p}}_{i}$ with a false discovery rate procedure such as Benjamini and Hochberg (1995). Unfortunately, analyzing nonasymptotic false discovery rate of such two‐step procedures is significantly more involved since their adaptive testing threshold is a function of ${\bar{p}}_{1},\text{…},{\bar{p}}_{m}$, as opposed to α/|S| in the two‐stage Bonferroni procedure presented here. To the best of our knowledge, the only method that has provable finite‐sample false discovery rate control in this context has been proposed by Bogomolov and Heller (2018), and further investigation into the problem of optimizing the threshold for selection in this setting is warranted.

## CONFLICT OF INTEREST

The authors have declared no conflict of interest.

### OPEN RESEARCH BADGES

This article has earned an Open Data badge for making publicly available the digitally‐shareable data necessary to reproduce the reported results. The data is available in the Supporting Information section.

This article has earned an open data badge “**Reproducible Research**” for making publicly available the code necessary to reproduce the reported results. The results reported in this article could fully be reproduced.

## Supporting information


Supporting Information

## Data Availability

Data for this article are publicly available as part of the following R packages: MultiMed and statgenGWAS, available from Bioconductor and CRAN, respectively.

## PROOFS AND TECHNICAL DETAILS

### A.1 Proof of Lemma 1

Consider first the distribution of the minimum ${\underline{p}}_{i}$ (to simplify notation, we omit the index i in what follows):

(A1)
$$P\left(\underline{p}\leq c\right)=1-P\left(\underline{p}>c\right)=1-P\left(p_{1}>c,p_{2}>c\right)=1-\prod_{j=1}^{2}P\left(p_{j}>c\right).$$

The joint distribution of $\bar{p}$ and $\underline{p}$ is

(A2)
$$P\left(\bar{p}\leq u,\underline{p}\leq c\right)=P\left(\bar{p}\leq u\right)=\prod_{j=1}^{2}P\left(p_{j}\leq u\right)$$

for 0<u≤c≤1, and

(A3)
$$\begin{array}{ccc} P(\bar{p}\leq u,\underline{p}\leq c) & = & P\left(\bar{p}\leq c\right)+P\left(\underline{p}\leq c,c<\bar{p}\leq u\right)\\ & = & \prod_{j=1}^{2}P\left(p_{j}\leq c\right)+\sum_{j=1}^{2}P\left(p_{j}\leq c\right)\left\{P\left(p_{-j}\leq u\right)-P\left(p_{-j}\leq c\right)\right\} \end{array}$$

for 0<c<u≤1, where $p_{-j}$ is $p_{2}$ for j=1, and $p_{1}$ for j=2.

The distribution of $\bar{p}$ conditional on the hypothesis $H_{i}$ being selected is $P(\bar{p}\leq u|\underline{p}\leq c)$. If the hypothesis $H_{i}$ is true then at least one of the p‐values $p_{1}$ and $p_{2}$ is null and thus uniformly distributed. Without loss of generality, let $H_{i1}$ be true, so that $P(p_{1}\leq x)=x$. Let F be the distribution function of $p_{2}$, so that $P\left(p_{2}\leq x\right)=F\left(x\right)$. Then from (A1)

(A4)
$$P\left(\underline{p}\leq c\right)=1-\left(1-c\right)\left\{1-F(c)\right\}=c+F\left(c\right)-cF\left(c\right),$$

and similarly for the joint distribution from (A2) and (A3)

(A5)
$$P\left(\bar{p}\leq u,\underline{p}\leq c\right)=\left\{\begin{array}{c} uF(u),\;\text{for}\;0<u\leq c\leq 1,\\ uF(c)+cF(u)-cF(c),\;\text{for}\;0<c<u\leq 1. \end{array}\right.$$

From this expression (3) follows. To obtain the result of the (0,0) pair, it is sufficient to replace F(x) with x in the above expression.

### A.2 Proof of Proposition 1

Let $I_{0}$ denote the index set of true union hypotheses, that is, the index set of (0,0), (0,1), and (1,0) pairs. Consider the probability of making no false rejections conditional on the selection G. It is 1 if no hypothesis passes the selection, that is, if $\sum_{j=1}^{m}G_{j}=0$, and otherwise

(A6)
$$\begin{array}{ccc} P(V=0|G) & = & P\left(\cap_{i:G_{i}=1\wedge i\in I_{0}}I\left[{\bar{p}}_{i}\geq \frac{\alpha }{\sum_{j=1}^{m}G_{j}}\right]|G\right)\\ & \geq & P\left(\cap_{i:G_{i}=1}I\left[{\bar{p}}_{i}\geq \frac{\alpha }{\sum_{j=1}^{m}G_{j}}\right]|G\right), \end{array}$$

(A7)
$$\begin{array}{ccc} & = & \prod_{i:G_{i}=1}P\left({\bar{p}}_{i}\geq \frac{\alpha }{\sum_{j=1}^{m}G_{j}}|G\right)\\ & = & \prod_{i:G_{i}=1}P\left({\bar{p}}_{i}\geq \frac{\alpha }{1+\sum_{j\neq i}G_{j}}|G\right)\\ & = & \prod_{i:G_{i}=1}P\left({\bar{p}}_{i}\geq \frac{\alpha }{1+\sum_{j\neq i}G_{j}}|I\left[{\underline{p}}_{i}\leq c\right],\sum_{j\neq i}G_{j}\right)\\ & = & \prod_{i:G_{i}=1}\left\{1-P\left({\bar{p}}_{i}\leq \frac{\alpha }{\left|S\right|}|I\left[{\underline{p}}_{i}\leq c\right],\left|S\right|\right)\right\}\\ & \geq & {\left\{1-P_{0}\left(\frac{\alpha }{\left|S\right|},\;c\right)\right\}}^{\left|S\right|}. \end{array}$$

In (A6), equality holds when for a given G, all selected hypotheses are true. This is true for all G if and only if $I_{0}=\left\{1,\text{…},m\right\}$. In (A7), equality holds if further all hypotheses are either a (0,1) or a (1,0) type. The conditional familywise error rate can be found as Pr(V≥1∣G)=1−Pr(V=0∣G). The expression (7) for the unconditional familywise error rate is obtained by taking the expectation over |S|.

### A.3 Proof of Proposition 2

To reject $H_{i}$, two events need to occur: ${\underline{p}}_{i}$ needs to be below the selection threshold c, and ${\bar{p}}_{i}$ needs to be below the testing threshold α/|S|. The probability of rejecting $H_{i}$ conditional on |S| is then:

(A8)
$$\begin{array}{ccc} P\left({\underline{p}}_{i}\leq c,\;\;\;{\bar{p}}_{i}\leq \frac{\alpha }{\left|S\right|}\right) & = & P\left({\bar{p}}_{i}\leq c\right)\;\;+\;\;P\left({\underline{p}}_{i}\leq c,\;\;\;c<{\bar{p}}_{i}\leq \frac{\alpha }{\left|S\right|}\right)\\ & = & F^{2}\left(c\right)+2F\left(c\right)\left[F\left(\frac{\alpha }{\left|S\right|}\right)-F\left(c\right)\right], \end{array}$$

if α/|S|≥c, and

(A9)
$$P\left({\underline{p}}_{i}\leq c,\;\;\;{\bar{p}}_{i}\leq \frac{\alpha }{\left|S\right|}\right)=P\left({\bar{p}}_{i}\leq \frac{\alpha }{\left|S\right|}\right)=F^{2}\left(\frac{\alpha }{\left|S\right|}\right),$$

if α/|S|<c.

### A.4 Oracle threshold and familywise error rate constraint

Let $P_{1}\left(c\right)$ denote the objective function and g(c)≤α the constraint of the optimization problem (10) in the main text. We have

(A10)
$$P_{1}\left(c\right)=P\left({\bar{p}}_{i}\leq \frac{\alpha }{E\left|S(c)\right|},{\underline{p}}_{i}\leq c\right)=\left\{\begin{array}{cc} 2F\left(c\right)F\left(\frac{\alpha }{E\left|S(c)\right|}\right)-F^{2}\left(c\right) & \text{for}\;c\in \left.(0,\bar{c}\right.];\\ F^{2}\left(\frac{\alpha }{E\left|S(c)\right|}\right) & \text{for}\;c\in (\bar{c},1), \end{array}\right.$$

where $\bar{c}$ is the unique solution of the equation c=α/E|S(c)|, and

(A11)
$$g\left(c\right)=1-{\left\{1-P_{0}\left(\frac{\alpha }{E\left|S(c)\right|},c\right)\right\}}^{E\left|S(c)\right|},$$

where $P_{0}$ is given in (3) in the main text. We show that the threshold that maximizes $P_{1}$ under the constraint is the smallest threshold that satisfies the familywise error rate constraint. First, we will show that c satisfies the constraint if it belongs to an interval $\left(c^{*},1\right)$, where $c^{*}$ is defined below. We will then show that $c^{*}$ is well approximated by $\bar{c}$. But, since E|S(c)| is a nondecreasing function of c, according to (A10), $P_{1}$ is nonincreasing for $c>\bar{c}$, so that the threshold that maximizes $P_{1}$ under the constraint is approximately $\bar{c}\approx c^{*}$.

First‐order approximation of the familywise error rate constraint in (A11) states:

(A12)
$$E\left|S(c)\right|P_{0}\left(\frac{\alpha }{E(S(c))},c\right)\leq \alpha .$$

It is straightforward to check that when c is close to zero, (A12) does not hold, while for $c=\bar{c}$, where $\bar{c}$ solves c=α/E|S(c)|, the constraint is satisfied. Namely, for $\bar{c}$ the selection threshold and the testing threshold coincide and according to (3) we have

(A13)
$$P_{0}\left(c,c\right)=c\;\;\frac{F(c)}{F\left(c\right)+c\left\{1-F(c)\right\}}\leq c$$

for all c∈(0,1), with equality holding if and only if F(c)=1. Given the continuity of $P_{0}$, this implies that there is a value $c^{*}$ in $\left(0,\bar{c}\right)$ such that the constraint holds with the equality. We now show that $c^{*}$ will be close to $\bar{c}$.

Denote $u_{c}=\alpha /E\left|S\left(c\right)\right|$. The equation $P_{0}\left(u_{c},c\right)=u_{c}$ simplifies to $F\left(u_{c}\right)-F\left(c\right)=u_{c}\left\{1-F\left(c\right)\right\}$ according to (3) since $c<u_{c}$. When m is large, the interval $\left(0,\bar{c}\right)$ will be small, and if we assume that F is locally linear in the neighborhood of c, we can substitute $F\left(u_{c}\right)\approx F\left(c\right)+f\left(c\right)\left(u_{c}-c\right)$, where f(·) is the density associated to F, to obtain

(A14)
$$u_{c}\approx c\;\;\frac{f(c)}{f(c)+F(c)-1}.$$

Since the density is strictly decreasing, for small values of c, |f(c)|≫|F(c)−1|, so that the above equation becomes

(A15)
$$u_{c}\approx c\;\text{i.e.}\;\alpha /E\left|S(c)\right|\approx c.$$

Therefore, the smallest threshold that satisfies the familywise error rate constraint can be approximated by $\bar{c}$.

### A.5 Proof of Theorem 1

We have

(A16)
$$\begin{array}{ccc} E(V) & = & E\sum_{i\in I_{0}}I\left[{\bar{p}}_{i}\leq c\right]=\sum_{i\in I_{0}}P\left({\bar{p}}_{i}\leq c\right)=\sum_{i\in I_{0}}P\left({\bar{p}}_{i}\leq c,{\underline{p}}_{i}\leq c\right)\\ & = & \sum_{i\in I_{0}}P\left({\bar{p}}_{i}\leq c|{\underline{p}}_{i}\leq c\right)P\left({\underline{p}}_{i}\leq c\right)\leq \sum_{i\in I_{0}}cP\left(\underline{p_{i}}\leq c\right)\\ & = & cE\sum_{i\in I_{0}}I\left[{\underline{p}}_{i}\leq c\right]\leq cE\sum_{i=1}^{m}I\left[{\underline{p}}_{i}\leq c\right]\\ & = & cE\left|S(c)\right|=\hat{PFER}. \end{array}$$

### A.6 Proof of Theorem 2

We have as in A.5

(A17)
$$\begin{array}{ccc} E(V) & = & E\sum_{i\in I_{0}}I\left[{\bar{p}}_{i}\leq c_{k}\right]=\sum_{i\in I_{0}}P\left({\bar{p}}_{i}\leq c_{k}\right)=\sum_{i\in I_{0}}P\left({\bar{p}}_{i}\leq c_{k},{\underline{p}}_{i}\leq c_{k}\right). \end{array}$$

Define

(A18)
$$c_{k}^{i}=\max\left\{c\in \left\{\frac{k}{m},\text{…},\frac{k}{2},k\right\}:c\;(1+\sum_{j\neq i}I\left[{\underline{p}}_{j}\leq c\right])\leq k\right\}.$$

Then, $c_{k}^{i}\leq c_{k}$ with equality if and only of ${\underline{p}}_{i}\leq c_{k}^{i}$. Furthermore, $c_{k}^{i}$ is independent of $\left({\underline{p}}_{i},{\bar{p}}_{i}\right)$. Let C denote the set $\left\{\frac{k}{m},\text{…},\frac{k}{2},k\right\}$. We can then write:

(A19)
$$\begin{array}{ccc} E(V) & = & \sum_{i\in I_{0}}\sum_{c\in C}P\left({\bar{p}}_{i}\leq c_{k},{\underline{p}}_{i}\leq c_{k},c_{k}=c\right)\\ & = & \sum_{i\in I_{0}}\sum_{c\in C}P\left({\bar{p}}_{i}\leq c_{k},{\underline{p}}_{i}\leq c_{k},c_{k}^{i}=c\right)\\ & = & \sum_{i\in I_{0}}\sum_{c\in C}P\left({\bar{p}}_{i}\leq c,{\underline{p}}_{i}\leq c|c_{k}^{i}=c\right)P\left(c_{k}^{i}=c\right)\\ & = & \sum_{i\in I_{0}}\sum_{c\in C}P\left({\bar{p}}_{i}\leq c,{\underline{p}}_{i}\leq c\right)P\left(c_{k}^{i}=c\right)\\ & = & \sum_{i\in I_{0}}\sum_{c\in C}P\left({\bar{p}}_{i}\leq c|{\underline{p}}_{i}\leq c\right)P\left({\underline{p}}_{i}\leq c\right)P\left(c_{k}^{i}=c\right)\\ & \leq & \sum_{i\in I_{0}}\sum_{c\in C}cP\left({\underline{p}}_{i}\leq c\right)P\left(c_{k}^{i}=c\right)\\ & = & \sum_{c\in C}c\sum_{i\in I_{0}}P\left({\underline{p}}_{i}\leq c\right)P\left(c_{k}^{i}=c\right)\\ & = & E\left(c_{k}^{i}\sum_{i\in I_{0}}I\left[{\underline{p}}_{i}\leq c_{k}^{i}\right]\right)\\ & \leq & E\left(c_{k}\sum_{i\in I_{0}}I\left[{\underline{p}}_{i}\leq c_{k}\right]\right)\\ & \leq & E\left(c_{k}\sum_{i=1}^{m}I\left[{\underline{p}}_{i}\leq c_{k}\right]\right)=E\left(c_{k}\left|S(c_{k})\right|\right)\leq k, \end{array}$$

where the second equality follows from the fact then when ${\underline{p}}_{i}\leq c_{k}$ then $c_{k}=c_{k}^{i}$.
