# Supplementary figures and images for: On optimal two‐stage testing of multiple mediators
Source: Biom J. 2022 Apr 14;64(6):1090–108. doi: 10.1002/bimj.202100190 (PMC9544827; doi:10.1002/bimj.202100190)

$m = 200$ , equal SNR

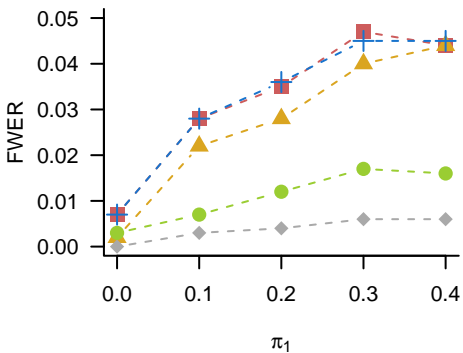

$m = 200$ , unequal SNR

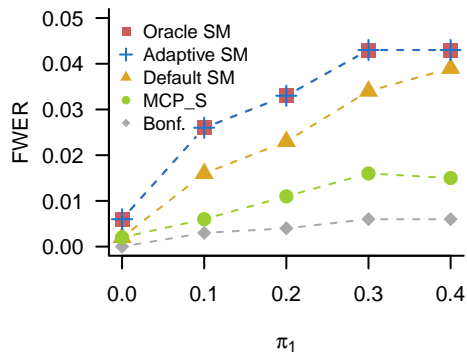

$m = 10000$ , equal SNR

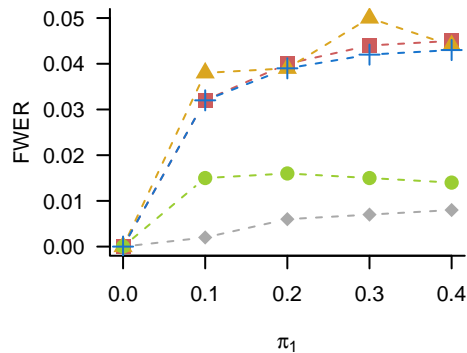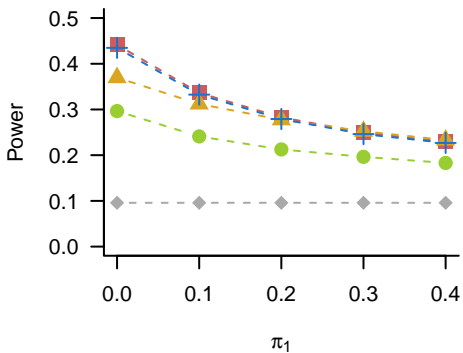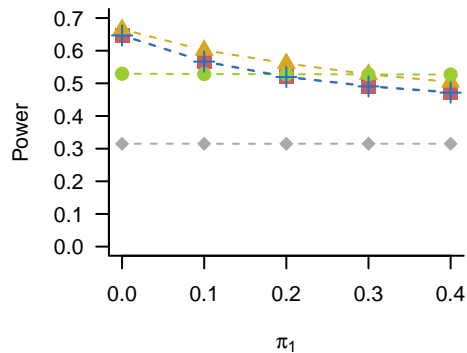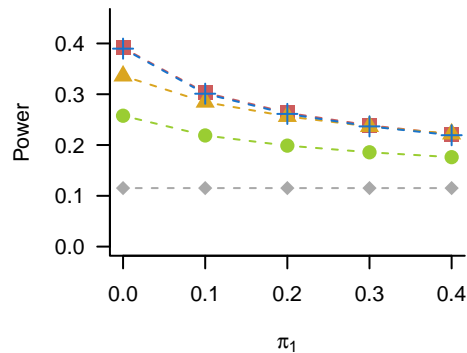

Supplement: Supplementary file 1 — Supporting Information [file BIMJ-64-1090-s001.zip › Code/results/Figure3_check.pdf]

QQ plot

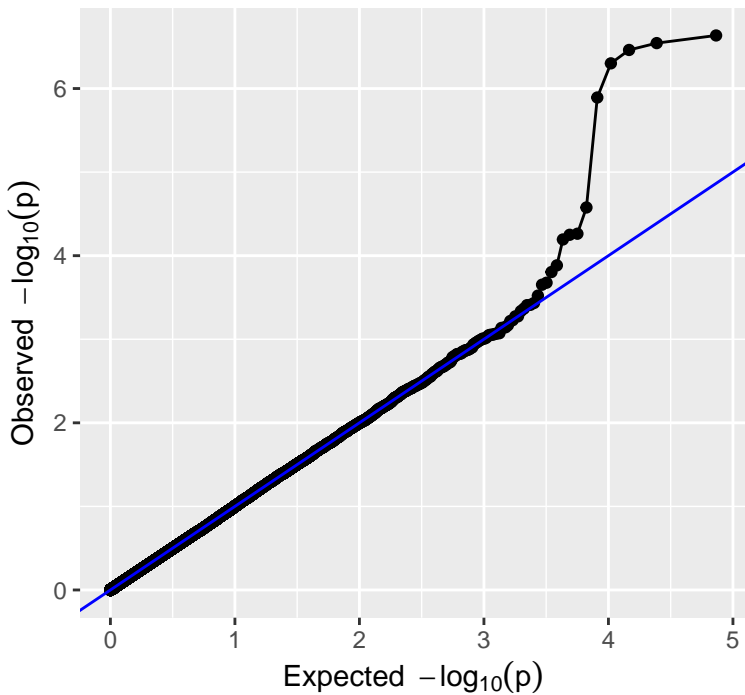

Supplement: Supplementary file 1 — Supporting Information [file BIMJ-64-1090-s001.zip › Code/results/qqplotMur13W.pdf]

QQ plot

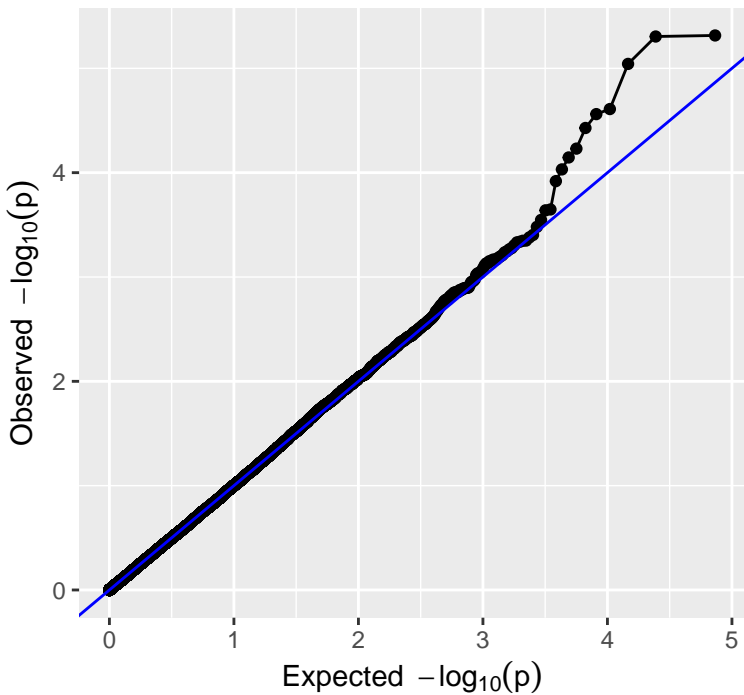

Supplement: Supplementary file 1 — Supporting Information [file BIMJ-64-1090-s001.zip › Code/results/qqplotKar13W.pdf]

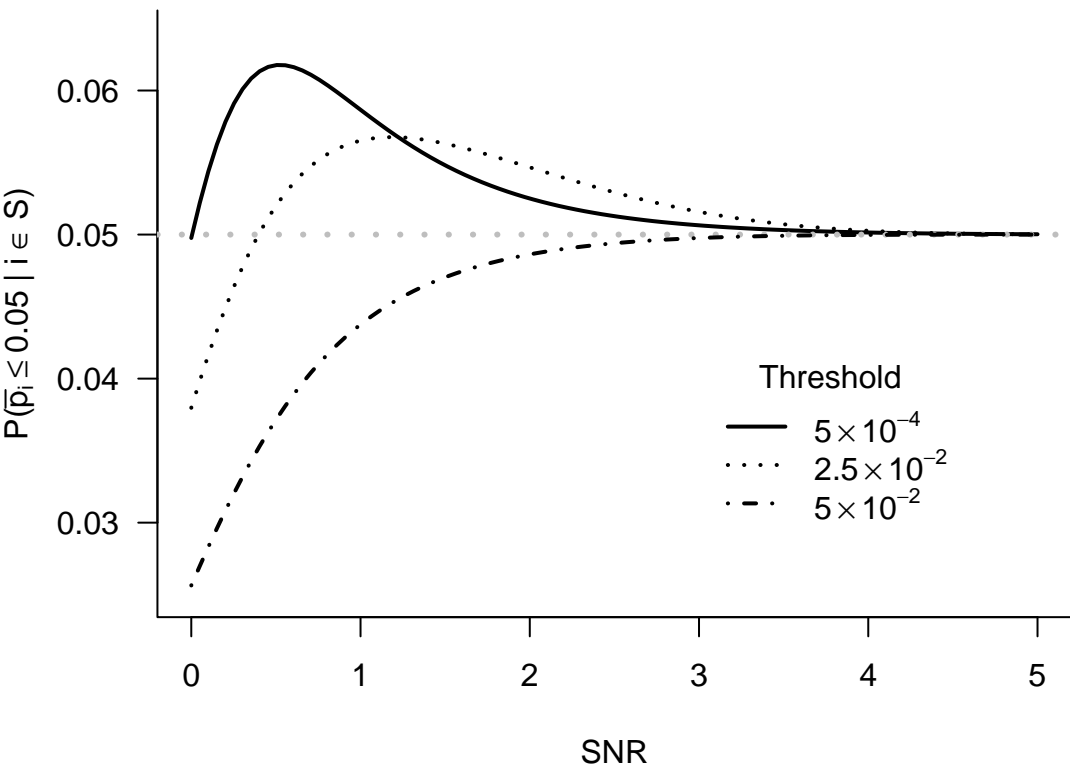

Supplement: Supplementary file 1 — Supporting Information [file BIMJ-64-1090-s001.zip › Code/results/Figure1.pdf]

**SNR = 1.5**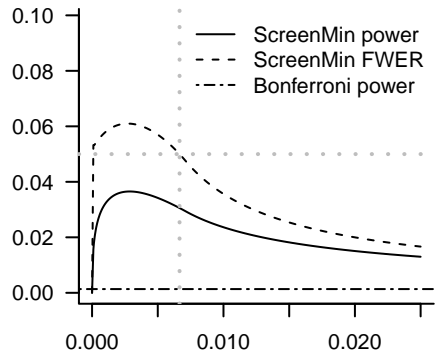 $c$ **SNR = 2**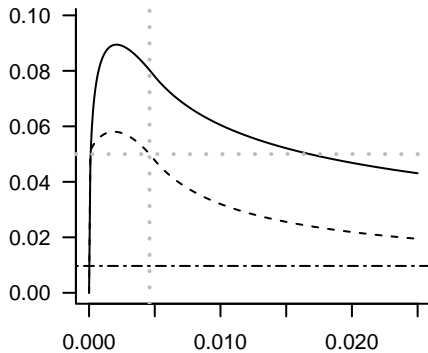 $c$ **SNR = 3**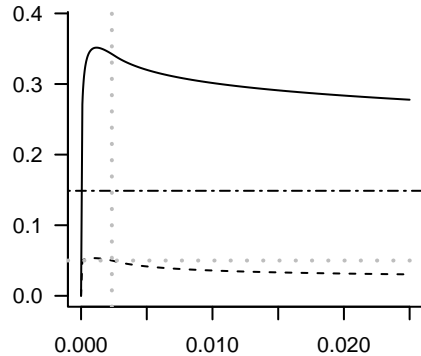 $c$

Supplement: Supplementary file 1 — Supporting Information [file BIMJ-64-1090-s001.zip › Code/results/Figure2.pdf]

$m = 200$ , equal SNR

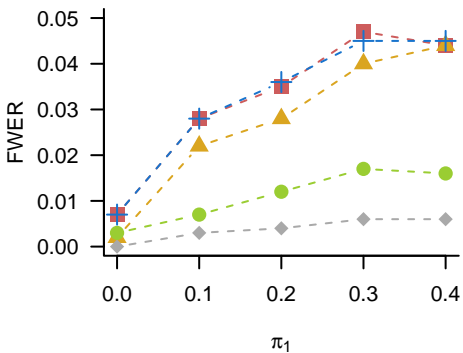

$m = 200$ , unequal SNR

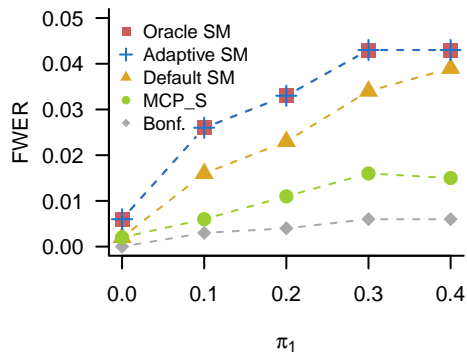

$m = 10000$ , equal SNR

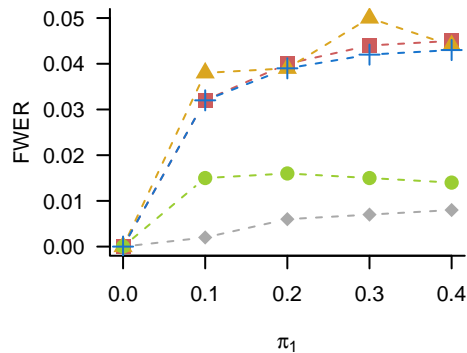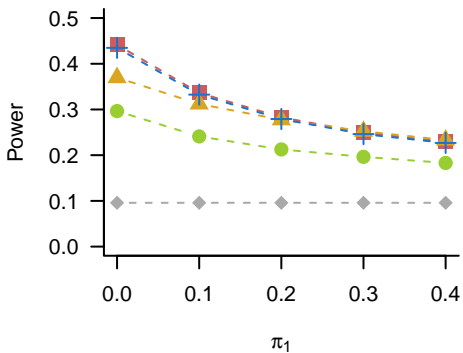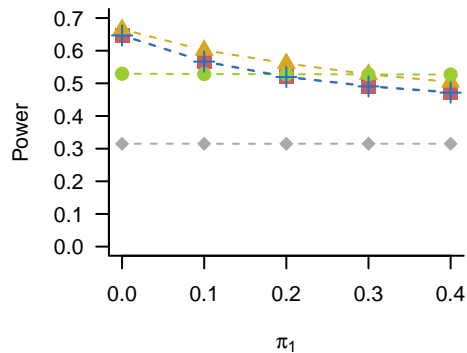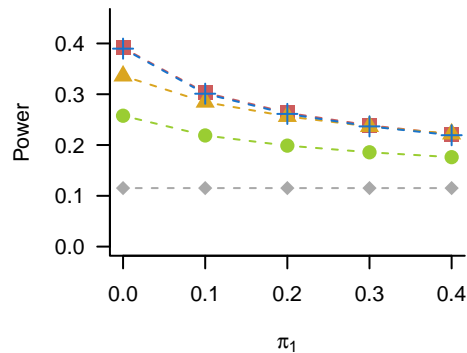

Supplement: Supplementary file 1 — Supporting Information [file BIMJ-64-1090-s001.zip › Code/results/Figure3.pdf]

$m = 200, \rho = 0.3$ , equal SNR

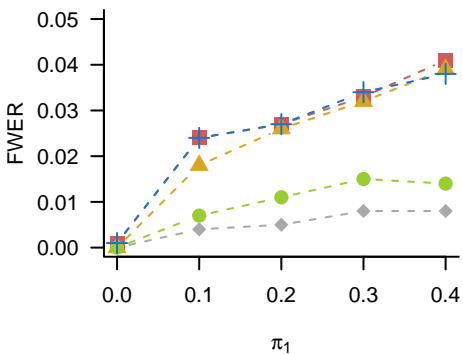

$m = 200, \rho = 0.8$ , unequal SNR

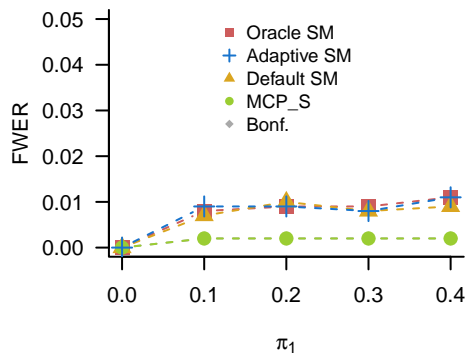

$m = 10000, \rho = 0.3$ , equal SNR

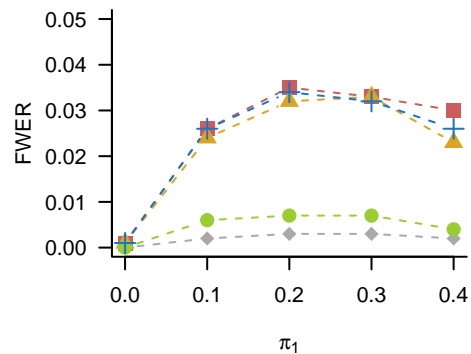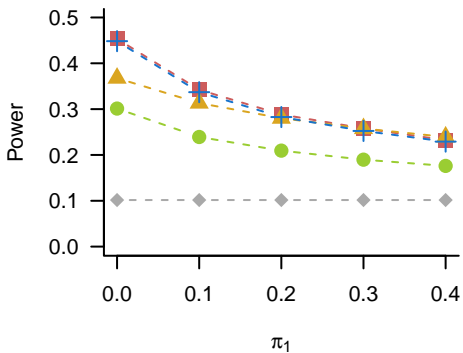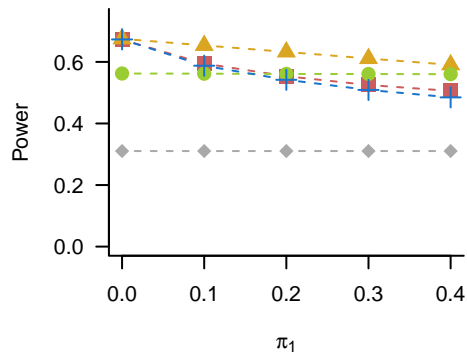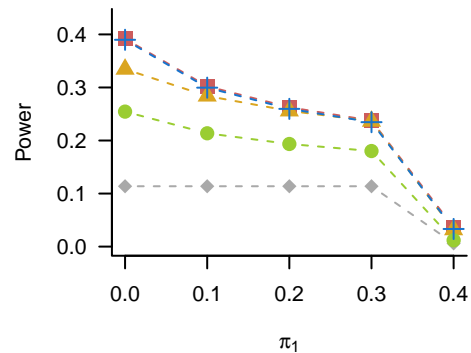

Supplement: Supplementary file 1 — Supporting Information [file BIMJ-64-1090-s001.zip › Code/results/Figure4.pdf]

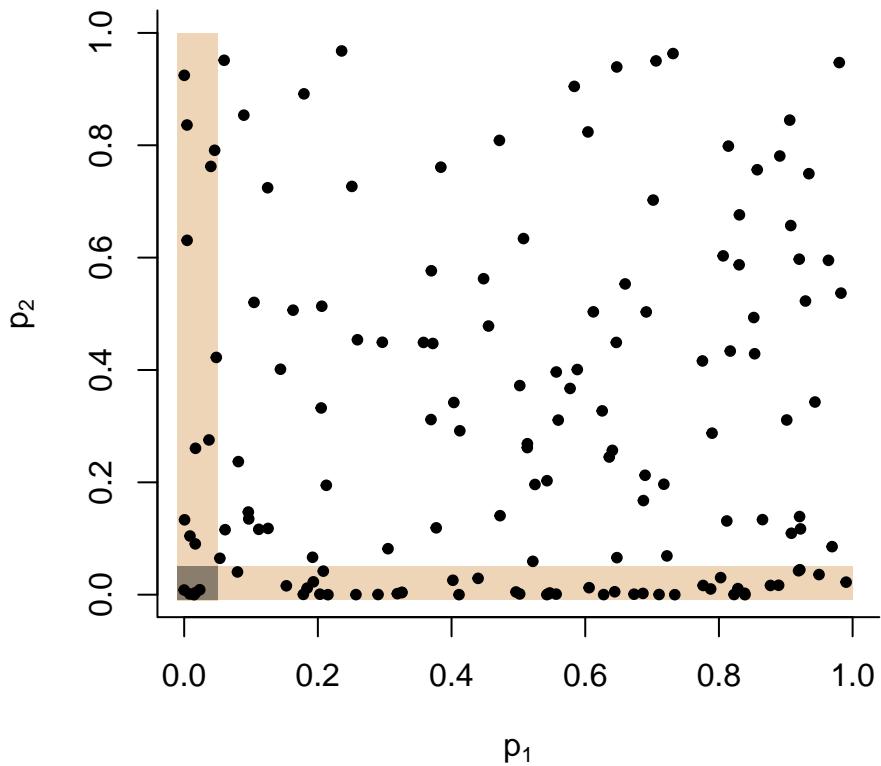

Supplement: Supplementary file 1 — Supporting Information [file BIMJ-64-1090-s001.zip › Code/results/Figure5.pdf]
